# Need of independent dose verification in brachytherapy

**DOI:** 10.4103/0971-6203.42743

**Published:** 2008

**Authors:** A Shanta

**Affiliations:** Retd. Scientific Officer, Ex. RP and AD, BARC, Mumbai, India E-mail: shanta.appukuttan@gmail.com

In brachytherapy, precise functioning of treatment planning system (TPS) in source localization and dose computations, using complex dose formalisms, is absolutely necessary to ensure planned dose delivery. Complex source geometry and the large number of foci involved in any implant make manual dose calculations very tedious. Hence manual dose verifications are rarely done and reported. Even during commissioning of a remote afterloading brachytherapy unit, where it is mandatory to incorporate manual dose verification of TPS output, almost all the users apply the simplest geometry, often suggested by the supplier. With advancement in treatment technology using stepping source and sophisticated dose optimization software, it has become a challenging task to verify optimized dose delivery to rule out any gross errors in TPS output. The Nuclear Regulatory Commission considers a difference of 20% between the prescribed total dose and administered total dose to be a reportable medical event.[1] Hence there is need for a quick method to verify the computational accuracy within 10%.[2]

The easiest approach to calculate the dose from a brachytherapy source is to consider the source to be a point source. To a reasonable accuracy, a source can be considered to be a point source if the distance of the point of dose computation is at least twice the active length of the source. Now, the most commonly used brachytherapy source is high dose rate (HDR) ^192^Ir,whose active length is less than 5 mm, for all the different types of units currently in use. Hence the minimum distance one can consider for dose verification is 10 mm. In case of long sources such as ^137^Cs tubes, the active length may be as much as 15 mm; but as these sources are used for gynecological intracavitary applications, a distance of 30 mm from the source will still be in the region of interest, clinically.

The total dose at a point is a function of distance of each source from the point of interest and the duration of treatment in each source position. In case of discrete sources with different activities, as in the case of Manchester type loading, as the duration of each source is same, it is the source strength which is the varying parameter. The distance between the center of a source and the point of interest can be easily calculated, knowing the three-dimensional coordinates of these points, read from TPS. If X, Y, Z are the coordinates of the point of interest (A) and Xi, Yi, Zi are the coordinates of the center of i^th^ source, distance (ri) of point A from this source is given by, ${\mathrm{ri}}^{2}=\left\{{\left(X-\mathrm{Xi}\right)}^{2}+{\left(Y-\mathrm{Yi}\right)}^{2}+{\left(Z-\mathrm{Zi}\right)}^{2}\right\}$ Dose at point A, using each source, may be calculated either using TG-43 parameters,

(1) $$\mathrm{i.e.},D_{\mathrm{Ai}}=\mathrm{Sk}.\wedge .\left(g(\mathrm{ri}).\mathrm{ti})/({\mathrm{ri}}^{2}\right)$$

or from basic principles,

(2) $$\mathrm{i.e.},D_{\mathrm{Ai}}=\mathrm{Sk}.\;(f_{\mathrm{ak},\mathrm{wat}})\;.\mathrm{WPF}(\mathrm{ri})\;\mathrm{ti}/({\mathrm{ri}}^{2})$$

where ‘Sk’ is the air kerma strength, ‘∧’ is the dose rate constant, ‘ti’ is the duration of treatment, g(ri) is the radial dose function, and WPF(ri) is the water perturbation factor (also referred to as Tissue Air Ratio). (f_ak,wat_) is the air kerma to dose in water conversion factor and is approximately 1.11 for ^192^Ir and ^137^Cs gamma energies.[3] The main difference between g(r), used in TG-43 formalism, and WPF(r) is that g(r) is the dose in water, normalized at 1 cm, along the perpendicular bisector of the actual source whereas WPF(r) is the ratio of dose in water to the dose in air for a point isotropic source. Both g(r) and WPF(r) vary only marginally with distance from the source up to about 5 cm for ^192^Ir gamma energies, and may be ignored for manual dose verification. For ^137^Cs, WPF is about 1.000 at 1.0 cm and decreases by about 1% per centimeter beyond this distance.

This method of dose verification may be utilized for inverse planning as well. In case of interstitial implants where often large numbers of sources are used, it is often not practical to do the manual calculation for all sources. In such cases, one may select an array of sources in actual implant and calculate the dose due to these sources alone at one reference point or more and compare with TPS-computed dose value/s. To verify the source localization accuracy of TPS, one may use a benchmark case. For example, consider a rectangular block of size 8 cm (length) × 6 cm (width) × 4 cm (thickness). The coordinates of the 8 corners of the block may be fed to the TPS by digitization of the anterior-posterior and lateral views of the block, drawn on a sheet of paper (preferably graph sheet). The manually derived coordinates of the corners of the block, with respect to the chosen origin and axes, may be compared with TPS-computed values. One may change the reference axes and the origin to anywhere — above, below, left, or right — and verify the modified three-dimensional coordinates of the corners of the block with respect to the new axes and origin.
